# Flow Cytometric Quantification of Peripheral Blood Cell β-Adrenergic Receptor Density and Urinary Endothelial Cell-Derived Microparticles in Pulmonary Arterial Hypertension

**DOI:** 10.1371/journal.pone.0156940

**Published:** 2016-06-07

**Authors:** Jonathan A. Rose, Nicholas Wanner, Hoi I. Cheong, Kimberly Queisser, Patrick Barrett, Margaret Park, Corrine Hite, Sathyamangla V. Naga Prasad, Serpil Erzurum, Kewal Asosingh

**Affiliations:** 1 Cleveland Clinic Lerner College of Medicine of Case Western Reserve University, Cleveland, Ohio, United States of America; 2 Department of Pathobiology, Lerner Research Institute, Cleveland Clinic, Cleveland, Ohio, United States of America; 3 Flow Cytometry Core, Lerner Research Institute, Cleveland Clinic, Cleveland, Ohio, United States of America; 4 Department of Molecular Cardiology, Lerner Research Institute, Cleveland Clinic, Cleveland, Ohio, United States of America; 5 Respiratory Institute, Cleveland Clinic, Cleveland, Ohio, United States of America; University of Louisville, UNITED STATES

## Abstract

Pulmonary arterial hypertension (PAH) is a heterogeneous disease characterized by severe angiogenic remodeling of the pulmonary artery wall and right ventricular hypertrophy. Thus, there is an increasing need for novel biomarkers to dissect disease heterogeneity, and predict treatment response. Although *β*-adrenergic receptor (βAR) dysfunction is well documented in left heart disease while endothelial cell-derived microparticles (Ec-MPs) are established biomarkers of angiogenic remodeling, methods for easy large clinical cohort analysis of these biomarkers are currently absent. Here we describe flow cytometric methods for quantification of βAR density on circulating white blood cells (WBC) and Ec-MPs in urine samples that can be used as potential biomarkers of right heart failure in PAH. Biotinylated β-blocker alprenolol was synthesized and validated as a βAR specific probe that was combined with immunophenotyping to quantify βAR density in circulating WBC subsets. Ec-MPs obtained from urine samples were stained for annexin-V and CD144, and analyzed by a micro flow cytometer. Flow cytometric detection of alprenolol showed that βAR density was decreased in most WBC subsets in PAH samples compared to healthy controls. Ec-MPs in urine was increased in PAH compared to controls. Furthermore, there was a direct correlation between Ec-MPs and Tricuspid annular plane systolic excursion (TAPSE) in PAH patients. Therefore, flow cytometric quantification of peripheral blood cell βAR density and urinary Ec-MPs may be useful as potential biomarkers of right ventricular function in PAH.

## Introduction

Pulmonary arterial hypertension (PAH) is a heterogeneous group of debilitating diseases characterized by hyperproliferative apoptosis-resistant pulmonary artery endothelial cells contributing to vascular remodeling of the small pulmonary arteries, which results in elevated pulmonary artery blood pressure and right ventricular failure [1–3]. *β*-adrenergic receptors (*β*AR) are transmembrane G-protein coupled receptors that are essential to many aspects of human physiology and are best known for control of cardiac chronotropy, inotropy, and vascular tone [4,5]. In left heart disease, *β*AR dysfunction results in progressive cardiac failure [4,6] and is associated with decreased *β*AR density in the cell membrane fraction of cardiac tissue [6–8] and circulating lymphocytes [9–11]. Importantly, previous studies have shown that reduced *β*AR density on the cell surface of circulating lymphocytes correlates directly with cardiac dysfunction in the failing left heart [10]. In that context, little is known about *β*AR density in circulating lymphocytes and its correlation to severity of PAH due to technical hurdles in the quantification of *β*AR density. Current methods for quantifying *β*AR density are laborious and time consuming that require radioactive detection of cell membrane extracts [9,10,12] which are not suitable for easy routine high throughput testing of patients.

Microparticles such as those derived from endothelial cells (Ec-MP) are vesicles generated by exocytic budding [13] and display surface antigens from their cell of origin [14]. Microparticles provide biological information about cell activation, cell injury, and apoptosis [15–18]. Despite the ability to glean this information from Ec-MPs, technical challenge of enumerating microparticles has prohibited its clinical utility. However with recent availability of micro flow cytometers, quantification and phenotyping of microparticles from blood or urine has become a possibility which offers the opportunity to assess Ec-MP as biomarkers of vascular remodeling [16,19]. Thus, our study here describes cytometric methods for the analysis of *β*AR density in peripheral blood cells and Ec-MPs in urine as potential biomarkers of right heart disease in patients with PAH.

## Materials and Methods

### Study population and sample collection

The study was approved by the institutional review board of the Cleveland Clinic, and written informed consent was obtained from all individuals in accordance with the Declaration of Helsinki. Patients with PAH and healthy volunteers between 18 and 65 years old with no history of asthma, insulin-dependent diabetes mellitus, thyroid disease, heart disease, and use of β-adrenergic agonist/antagonist in the last three months were enrolled. Peripheral venous blood was collected in EDTA tubes and processed within two hours. Spot urine samples were collected and processed within one hour. Basic demographic and clinical information was collected from healthy volunteers using an enrollment questionnaire.

### Tricuspid annular plane systolic excursion (TAPSE) Measurement

TAPSE was measured on all PAH patients according to the latest American Society of Echocardiography Right Ventricle Chamber Guidelines [20]. TAPSE was applied to assess right ventricle global systolic function (systolic and diastolic) using M-mode at the tricuspid valve annulus in the apical 4-chamber view, allowing measurement of the longitudinal motion of the annulus. TAPSE assumes that the systolic motion of the tricuspid annulus is representative of the function of the entire right ventricle. Values equal to or less than 1.6 cm distinguishes abnormal from normal right ventricle function. TAPSE may not be accurate in subjects with right ventricle regional wall motion abnormalities of the lateral right ventricle free-wall. Additional limitations of TAPSE are that it is angle dependent and may be load dependent. TAPSE has been validated against other measures of right ventricle global function (radionuclide angiography, RV percent fractional shortening, and Simpsons right ventricle ejection fraction).

### Isolation and processing of peripheral blood cells from whole blood

Peripheral blood cells were isolated from 12 mL of whole blood as described previously [21,22]. In short, blood was aliquoted into 4 mL samples and fixed using 10% formaldehyde, followed by permeabilization and lysis of red blood cells (RBC) in a single step using Triton X100 at a final concentration of 0.2%. After a series of washes with 4% FBS in PBS, peripheral blood cells were divided into aliquots of approximately 10 x10^6^ cells and frozen at -80°C in freezing medium consisting of 10% glycerol, 20% FBS in RPMI 1640.

### βAR flow cytometry assay

Peripheral blood cells were analyzed for various cell subpopulations by using anti–human CD3 PE/Cy7 and CD19 PE/Cy5 (Biolegend, San Diego, CA), CD45 Alex Fluor 700 (eBioscience, San Diego, CA), CD133/1 (AC133) APC (Miltenyi Biotec, Auburn, CA), CD34 FITC (BD Biosciences, San Jose, CA), and 4′,6-diamidino-2-phenylindole (DAPI) (Life Technologies, Grand Island, NY) (Table 1). Peripheral blood cells subsets were then stained with a custom-made probe alprenolol-biotin, which consists of the β-blocker alprenolol covalently linked to biotin (Cell Mosaic, Worcester, MA) (S1 Fig). The alprenolol probe was detected by secondary reagent Streptavidin PE (eBioscience). Cells were thawed to room temperature and pre-incubated with 10% goat serum, 1:40 Trustain FcX (Biolegends) to block nonspecific Fc-receptor antibody binding, followed by incubation with 10 μg/mL unconjugated Streptavidin (Life Technologies) to block nonspecific binding of Streptavidin PE to endogenous biotin. Cells were then stained for DAPI, followed by anti-human CD3, CD19, CD45, CD34 and CD133. In subsequent steps, cells were stained with alprenolol-biotin probe followed by streptavidin PE. Compensation was accomplished for each acquisition using single color labeled peripheral blood cells stained with anti-human CD45 FITC, CD45 APC, and CD45 PE (eBioscience), CD3 PE/Cy7, CD19 PE/Cy5, CD45 Alex Fluor 700, and DAPI. Six peak SPHERO Ultra Rainbow Calibration Kit (Spherotech, Lake Forest, IL) was used to standardize fluorescence detectors for day to day variation. At least 50,000 events for unstained tubes, 400,000 events for (fluorescence minus one) FMO tubes, and 1,250,000 events for stained tubes were acquired on a Fortessa (Becton Dickinson) flow cytometer equipped with 5 lasers (355nm, 407nm, 488nm, 561nm and 641nm) and data was saved as listmode files. Data analysis was performed using FlowJo software (vX.0.7) (Tree Star, OR). Alprenolol binding was expressed as the median fluorescence intensity (MFI) of each cell subset. A reference sample run with each batch was used to standardize across different runs.

**Table 1:** Antibodies and probes used for flow cytometric assay.

Antibody or Probe	Vendor	Isotype	Dilution or Concentration
DAPI	Life Technologies	N/A	1/400
CD3 PE/Cy7	Biolegend	Mouse IgG_1_	1/1600
CD19 PE/Cy5	Biolegend	Mouse IgG_1_	1/27
CD45 Alex Fluor 700	Biolegend	Mouse IgG_1_	1/800
CD34 FITC	BD Bioscience	Mouse IgG_1_	1/5.4
CD133/1 (AC133) APC	Miltenyi Biotec	Mouse IgG_1_	1/2.7
Alprenolol-Biotin	Cell Mosaic	N/A	25 μg/mL
Streptavidin PE	eBioscience	N/A	1/200
CD45 FITC	eBioscience	Mouse IgG_1_	1/20
CD45 APC	eBioscience	Mouse IgG_1_	1/20
CD45 PE	eBioscience	Mouse IgG_1_	1/100

### Endothelial cell microparticle flow cytometry

Spot urine samples were centrifuged at 900 g for 5 minutes in 50 mL conical tubes to remove debris and cells. Aliquots of supernatants were frozen at -80°C for batch analysis. Frozen samples were thawed at room temperature and centrifuged at 15,000 g for 5 minutes using the Eppendorf Centrifuge 5424 (used in all subsequent steps). Supernatants were aspirated down to the 100 μL mark on the eppendorf tube. Microparticles were washed by adding 900 μL of annexin V binding buffer (140mM NaCl, 10mM HEPES, 2.5 mM CaCl_2_, double filtered using 0.1 μM syringe filter (Minisart) to reduce background noise) followed by centrifugation at 15,000 g for 5 minutes and aspirated. Aliquots of 5 μL per tube were used for staining. Vascular endothelial (VE) cadherin, also known as CD144, is an antigen that is located at endothelial cell junctions and is expressed exclusively by endothelial cells, making it the most specific endothelial cell marker. Annexin V was used as a marker for microparticles. Tubes to set compensation for spectral overlap included unstained, stained with annexin-V-AlexaFluor 488 only, and stained with CD144-PE only. Unstained, annexin-V only, and annexin-V combined with endothelial cell marker CD144-PE tubes were run for each sample. Optimal dilutions for annexin-V-AlexaFluor 488 (1/20, Life Technologies) and CD144-PE (1/4 Santa Cruz Biotechnology) were determined by titrating the reagents on microparticles isolated from platelet free plasma. The following markers we used to detect hematopoietic cell-derived microparticles: CD3-PE (eBiosciences), CD19-PE (eBiosciences), CD45-PE (eBiosciences), CD34-PE (Becton Dickinson) and CD133 (Miltenyi Biotec), all dilutions 1/100 in a final labeling volume of 100 μL. Isotype matched control IgG-PE (Becton Dickinson) were run to control for unspecific antibody binding. Elimination of antibody aggregates and verification of true microparticles and optimal microparticle concentration to prevent coincidence were performed according to current guidelines [23,24]. Reagent vials were centrifuged at 15,000 g for 10 minutes to pellet potential aggregates prior to pipetting aliquots for staining. Presence of true microparticles was further validated by dissolving vesicles by treating samples with Triton-X 100 detergent. All reagents were diluted in annexin V binding buffer and incubations were performed for 30 minutes at room temperature. Unstained samples underwent the same washings as stained samples. S2 Fig shows an outline of the sample processing method for staining. Samples were analyzed using the Apogee A50 Micro Flow Cytometer (Apogee, Hertfordshire, UK). Instrument’s sensitivity, calibration and standardization strategy are shown in S3 Fig. Samples were acquired at a flow rate of 6.01 μL/min for 2 minutes. At least 5000 annexin-V^+^ CD144-PE^+^ microparticles were acquired for each sample.

### Statistical analysis

Results were analyzed using Excel (Microsoft, Redmond, WA) and JMP Pro 10 (SAS, Cary, NC) software programs. Descriptive statistics were reported for each study group and peripheral blood cell subpopulation. Student’s t test was used to compare continuous variables such as MFI alprenolol-biotin binding and %CD144^+^ microparticles among the two study populations. Spearman’s correlation coefficient was used to correlate the MFI of subsets and %CD144^+^ microparticles with the continuous clinical variable of TAPSE. *P*< 0.05 was considered significant.

## Results

### Study population

Twenty healthy controls (CTRL) and 22 PAH patients were enrolled in this study. Demographics of the study groups and clinical parameters of PAH patients are shown in Table 2. Mean age of patients was 40.7 ± 10.7 years for CTRL and 45.2 ± 11.5 years for PAH, *p* = 0.10. Gender of patients was 60% female for CTRL and 59% for PAH, *p* = 0.95. Race of patients was mostly Caucasian including 85% for CTRL and 82% for PAH, *p* = 0.45. There were no statistically significant differences in demographics between the study groups. Clinical parameters of PAH patients included mild to moderate severity with 73% in New York Heart Association functional class II (I/II/III/IV: 2/18/4/0), mean 6-minute walk distance of 1590 feet, and right ventricular systolic pressure of 64.5 mmHg. Urine was collected from a subset of these study groups and demographics and clinical parameters are shown in S1 Table.

**Table 2:** Study population characteristics.

	Healthy Control	PAH	p-value*
**Number**	20	22	
**Age (years), mean ± SD**	40.0 ± 10.7	45.2 ± 11.5	0.10
**Gender (% female)**	60%	59%	0.95
**Race (C/AA/Other), N (%)**	17(85%)/2(10%)/1(5%)	18(82%)/4(18%)/0(0%)	0.45
**NYHA Class (I/II/III/IV), N (%)**	-	2(9%)/16(73%)/4(18%)/0(0%)	-
**6MWD (ft), mean ± SD**	-	1590 ± 380	-
**RVSP (mmHg), mean ± SD**	-	64.5 ± 27.2	-

*p-value represents Student’s t test comparison of study groups for continuous variables and Χ^2^ comparison for categorical variables. C = Caucasian, AA = African American.

### Alprenolol-biotin binding for evaluating β-adrenergic receptor density

**Fig 1:**
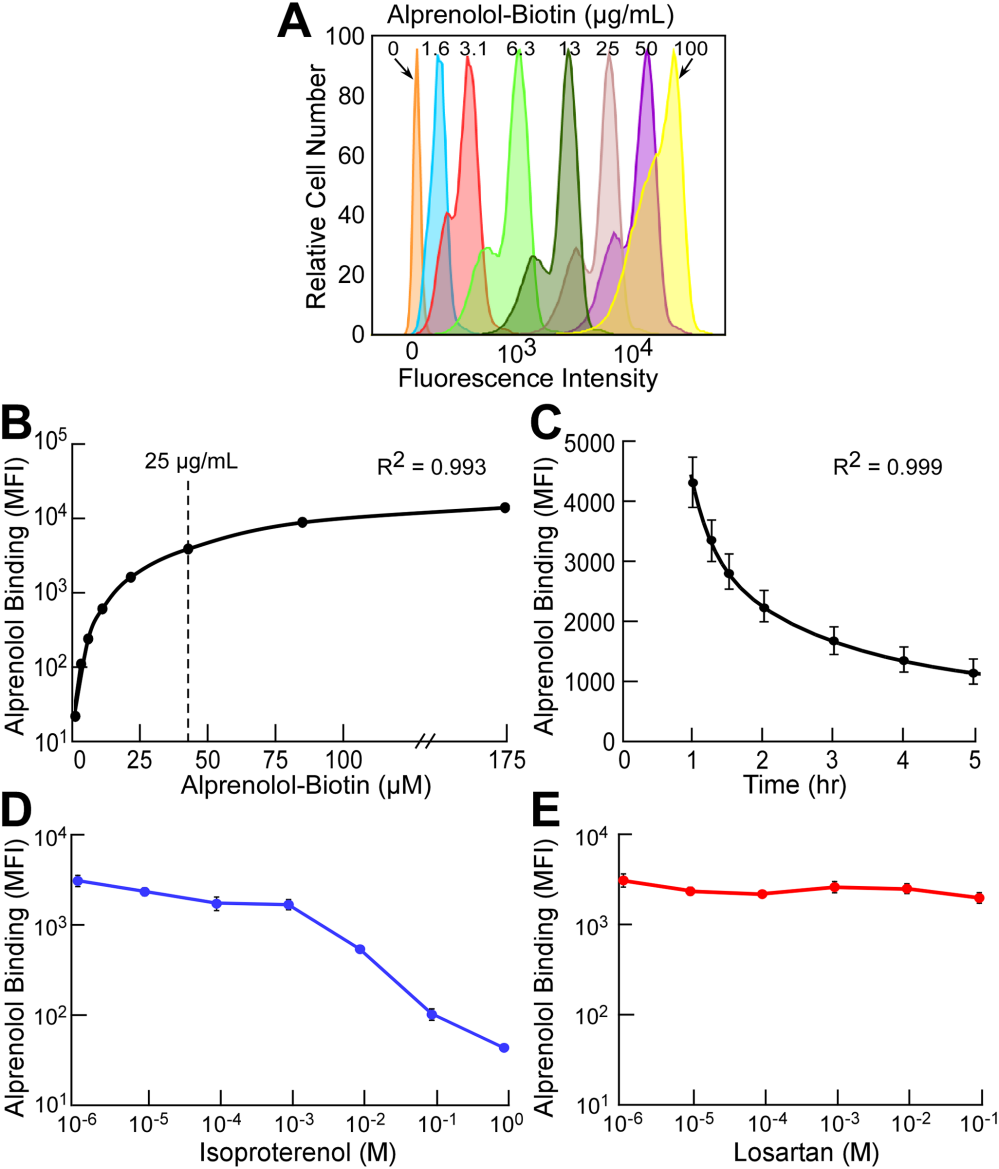
Alprenolol-biotin binding of human PBCs specific for the βAR. **A)** Titration of the alprenolol-biotin probe demonstrated an optimal concentration of 25 μg/mL. Values represent MFI for WBC population. **B)** The titration curve shows that binding is a saturable and specific process. **C)** Decay curve for alprenolol binding. Values represent mean ± SE of 2 experiments each with N = 3. **D)** Isoproterenol, another ligand for βAR, displaced alprenolol from the receptor in a dose-dependent fashion. **E)** Losartan, an angiotensin II receptor antagonist (scramble competitor) did not affect binding of the alprenolol probe. Mean ± SE of 2 experiments are shown.

Binding of the alprenolol-biotin probe to peripheral blood cells was first titrated to determine optimal concentration for specific binding and population resolution. As shown in Fig 1A, at a probe concentration of 25 μg/mL (43 μM), there was near-maximum saturation and good population resolution with minimal non-specific binding. The titration curve in Fig 1B shows that alprenolol-biotin binding was a saturable and specific process reminiscent of classic ligand binding to finite number of receptors. In addition, the binding of alprenolol was found to decay over time as previously reported [12,25], leading to use of correction for dissociation according to the decay curve allowing us to compare samples (Fig 1C).

Binding specificity of the alprenolol probe to βAR was confirmed with competition experiments in which cells labeled with alprenolol-biotin were exposed to increasing concentrations of cold βAR agonist isoproterenol or different G-protein coupled receptor ligand losartan, an angiotensin II receptor blocker, as a non-specific scramble competitor. Consistently, isoproterenol competed for alprenolol binding sites in a dose-dependent manner [12] (Fig 1D) while, non-specific competitor losartan did not alter the binding affinity of alprenolol (Fig 1E). Together these data demonstrate that binding of the alprenolol probe was specific for βAR.

### Gating subpopulations of peripheral blood cells

**Fig 2:**
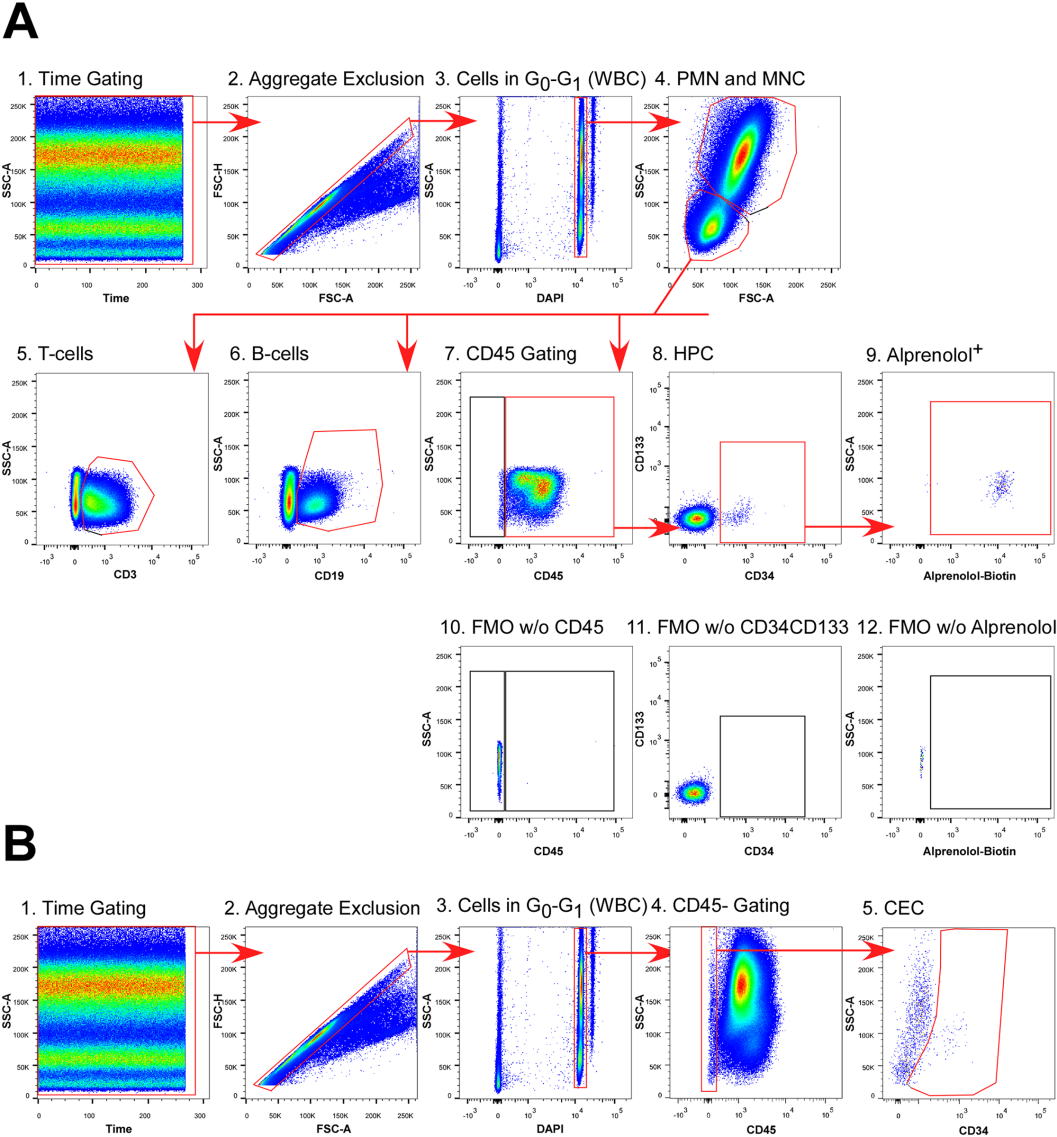
Gating strategy for flow cytometry assay. **A)** Gating strategy for hematopoietic peripheral blood cell subsets. Artifact exclusion included time gating (1), aggregate exclusion (2), and DAPI gating for cells in G_0_/G_1_ (3). FSC vs SSC gating was used to discriminate polymorphonuclear cells (PMN) and mononuclear cells (MNC) (4). CD3 was used as a T-cell maker (5) and CD19 as a B-cell marker (6). CD45 gating of the CD3^-^CD19^-^ MNC (**7**) based on fluorescence-minus one (FMO) control (**8**), followed by CD34 and CD133 gating (**9**) based on FMO control (**10**) were used to select hematopoietic progenitor cells (HPC). Alprenolol binding was then assessed based on FMO control (**11**). **B)** Gating strategy for circulating endothelial cells (CEC). Artifact exclusion included time gating (**1**), aggregate exclusion (**2**), and DAPI gating for cells in G_0_ and G_1_ (**3**). CD45^-^ gating of the CD3^-^CD19^-^ population was based on FMO control (**4**). CD34^+^ gating was used to define CEC (**5**).

Subsets of peripheral blood cells were selected from the parent population using the flow cytometric gating approach depicted in Fig 2 wherein a series of gating strategies were used to eliminate artifacts. A first set of gating included time gating to control for fluidic disturbances in the flow cell (Fig 2A1), aggregate exclusion (Fig 2A2), and DAPI^+^-gating of cells in the G_0_/G_1_ phase of the cell cycle (Fig 2A3) to select for true single-cell WBC events. Gating based on traditional forward scatter (FSC) vs side scatter (SSC) profiles were used to distinguish PMN and MNC populations (Fig 2A4). The MNC population of cells was then gated for CD3 and CD19 for T-cells (Fig 2A5) and B-cells (Fig 2A6), respectively. For hematopoietic progenitor cells (HPC) gating, CD3^+^ or CD19^+^ events were excluded and gated for CD45 (Fig 2A7) based on a fluorescence minus one (FMO) control lacking CD45 (Fig 2A10). CD133 and CD34 were then used to select HPC (Fig 2A8), which were CD34^+^CD133^lo/-^, based on a control missing CD34 and CD133 (Fig 2A11). Alprenolol^+^ cells were identified (Fig 2A9) using an FMO control missing the alprenolol probe (Fig 2A12). Circulating endothelial cells (CECs) were identified by CD45^-^ gating of the CD3^-^CD19^-^ WBC population (Fig 2B4) followed by gating of CD34^+^ cells (Fig 2B5). The median fluorescence intensity (MFI) of alprenolol-biotin was measured for each subpopulation to quantify total (cell surface and intracellular) βAR density. In this cohort there was a slight increase in percentage of PMN and decrease in MNC in PAH patients as compared to healthy controls. Furthermore, there was an increase in the percentage of circulating endothelial cells in PAH patients, which is consistent with previous findings [26,27] (Table 3).

**Table 3:** Percentage of PBC subsets in healthy controls (CTRL) and PAH patients.

PBC Subset	CTRL	PAH	
	Mean (%) ± SD (%)	Mean (%) ± SD (%)	p-value
PMN*	68.0 ± 7.51	73.1 ± 8.88	0.051
MNC*	30.9 ± 7.05	25.7 ± 8.79	**0.044**
**T-cell**^†^	67.9 ± 8.34	68.3 ± 7.99	0.878
**B-cell**^†^	11.3 ± 4.55	12.4 ± 4.71	0.426
**PAC**^‡^	0.57 ± 0.59	0.74 ± 0.69	0.398
**CEC**^§^	0.0086 ± 0.005	0.0122 ± 0.0063	**0.046**

*Represents percentage of WBC cell population as defined in methods section

^†^Represents percentage of MNC population as defined in methods section

^‡^Represents percentage of CD3^-^CD19^-^CD45^+^ MNC population as defined in methods section

^§^Represents percentage of CD3^-^CD19^-^ WBC population as defined in methods section

### β-adrenergic receptors in circulating cells of healthy controls and PAH patients

**Fig 3:**
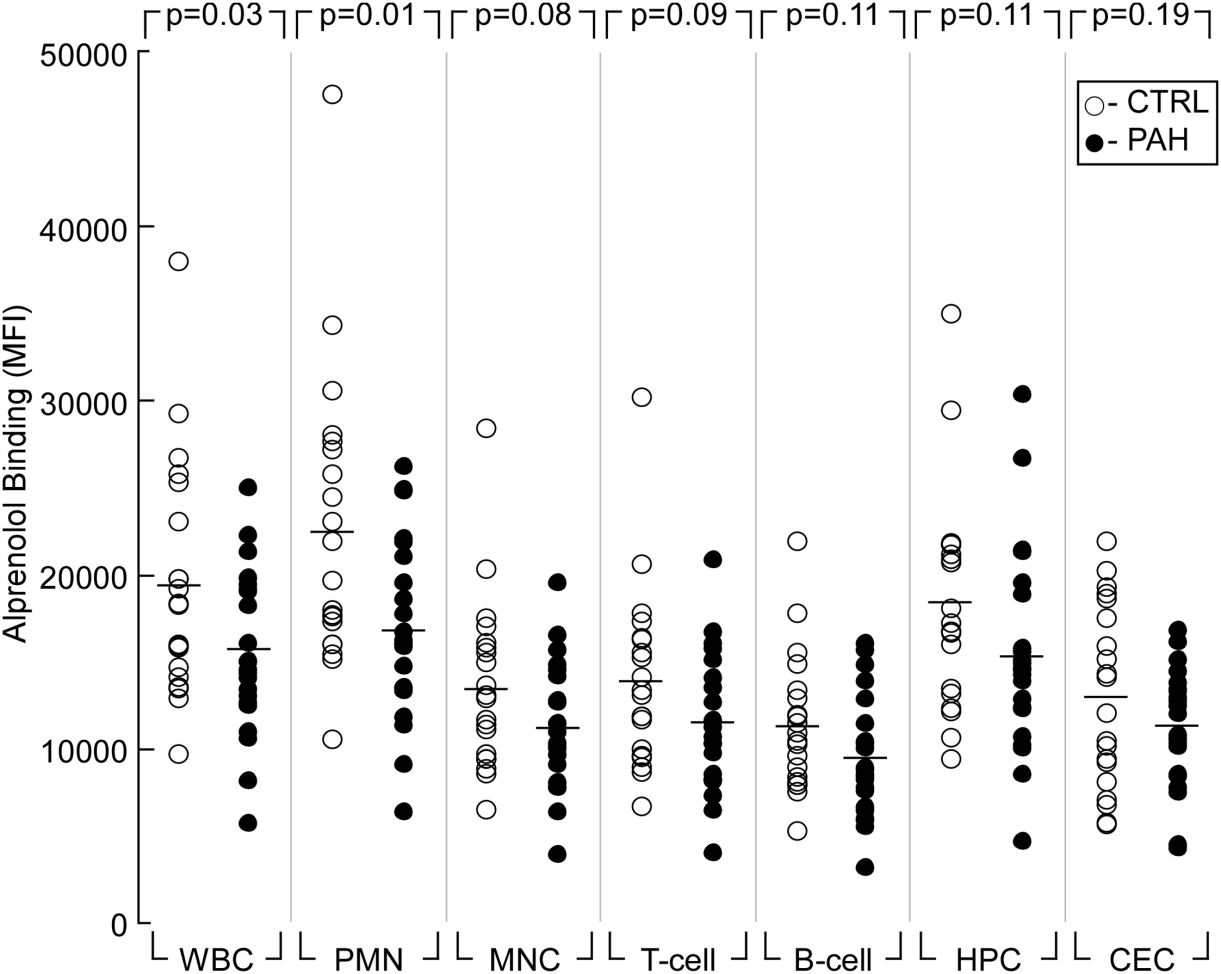
Alprenolol-biotin binding in subsets of peripheral blood cells is decreased in PAH as compared to controls. p-value represents Student’s t test comparison of study groups.

Flow cytometric analysis of alprenolol-biotin binding demonstrated that various peripheral blood cell subsets are characterized by differential levels of βAR expression (Fig 3). The profile of variability in βAR expression pattern between subsets appeared similar in healthy controls and PAH patients. PMN had the highest receptor expression in both healthy controls and PAH patients while B-cells had the lowest levels of binding [ANOVA comparing alprenolol binding for cell subsets: CTRL, PAH p<0.0001 (data not shown)]. Interestingly, HPC had the highest levels of βAR expression compared to other cell subsets of MNC (like T-cell, B-cell, and CEC) (t-test comparing MNC and HPC: CTRL p = 0.01; PAH p = 0.002). Analysis of patient cohorts within a given cell subset showed significant differences between controls and PAH patients with PAH patients having lower levels of βAR density in the total cell population (WBC p = 0.03) and PMN subset (p = 0.01) and a trend toward lower levels in the remaining subsets of cells (Fig 3).

### β-adrenergic receptor levels in PAH correlates with right ventricular function

**Fig 4:**
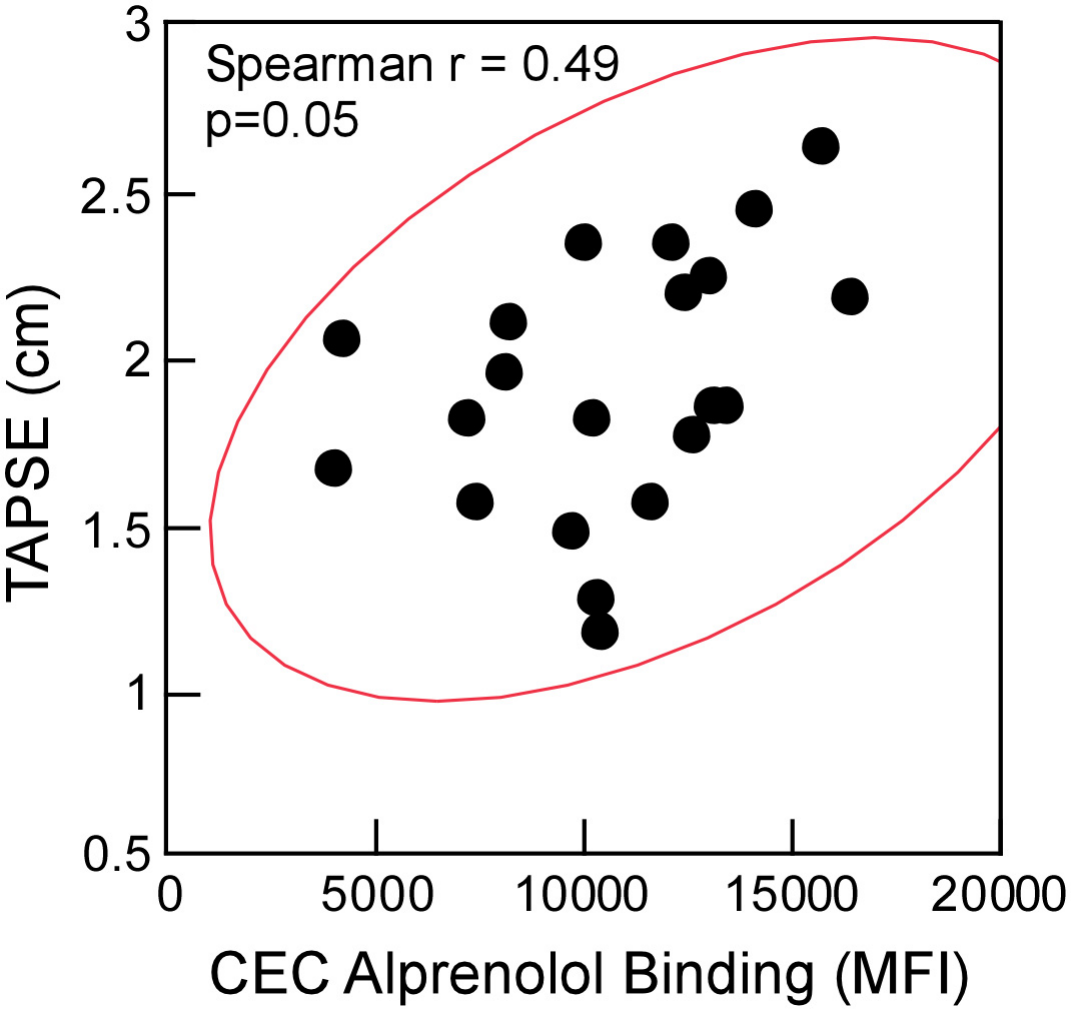
Alprenolol-biotin binding in circulating endothelial cells (CEC) correlates with TAPSE. ρ represents Spearman's rank correlation coefficient with corresponding p value.

Alprenolol-biotin binding was tested for correlation with Tricuspid annular plane systolic excursion (TAPSE) in PAH patients as measured by echocardiography at time of enrollment into the study. There was a trend towards a positive correlation of alprenolol binding and TAPSE. Higher levels of alprenolol binding ie. higher βAR density in the CEC subset correlated with high TAPSE measurement which is associated with better right ventricular function (Spearman’s test: ρ = 0.49, p = 0.05) (Fig 4). No correlation was observed between TAPSE and circulating hematopoietic subsets (S2 Table).

### Gating strategy for endothelial-derived vesicles

**Fig 5:**
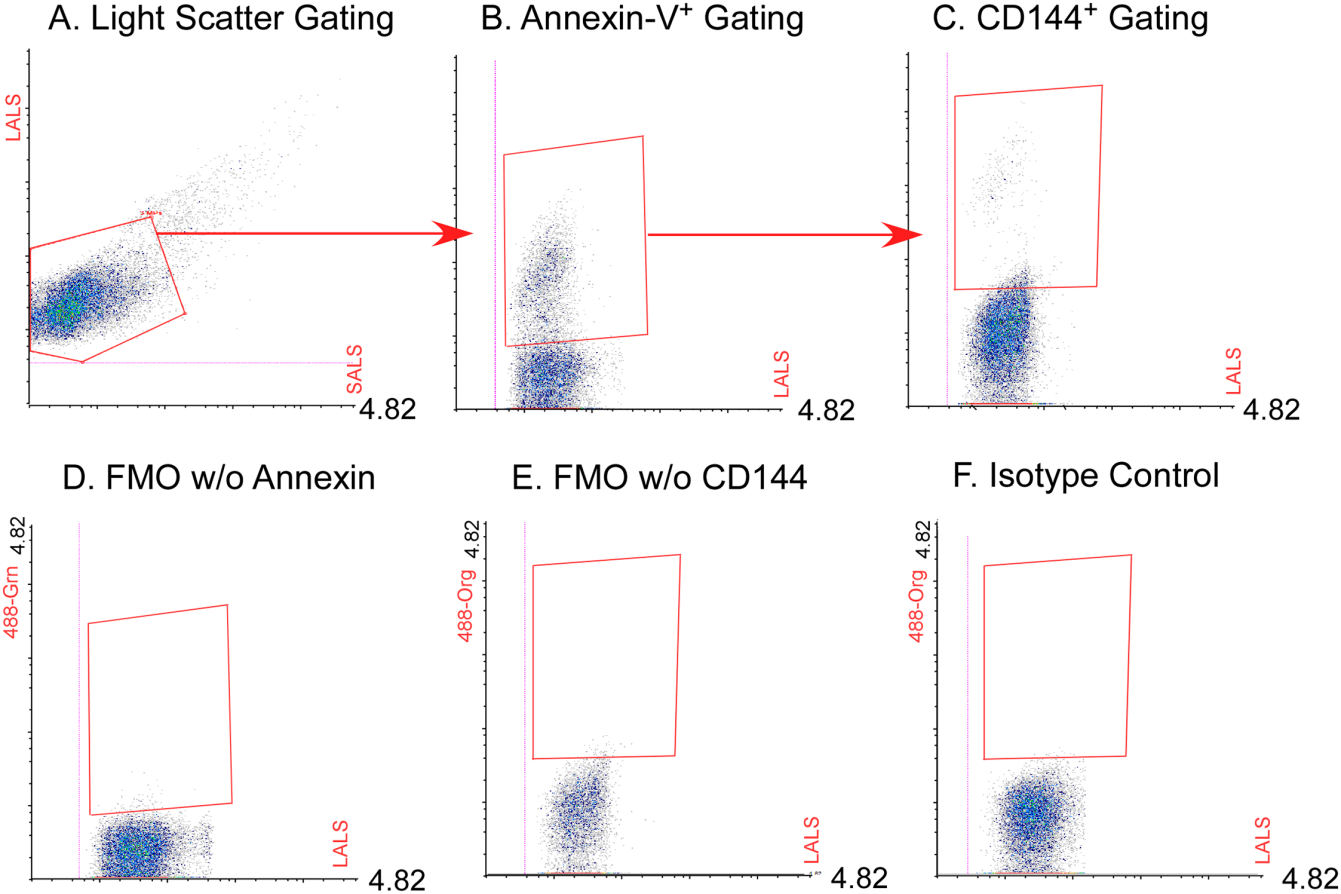
Gating strategy for urinary endothelial cell microparticles. Sequential gating was used to quantify the percentage of endothelial-derived microvesicles among annexin-V positive microparticles in the urine. Small Angle Light Scatter (SALS) and Large Angle Lights Scatter (LALS) were used to gate the microvesicle population (**A**). Annexin-V^+^ events were selected on a LALS/Green fluorescence channel plot (**B**). Annexin-V^+^ region was defined based on unstained sample (**D**). CD144 (VE-cadherin)^+^ events in the annexin-V^+^ gate were defined on an LALS/orange fluorescence channel (**C**) based on an FMO control missing CD144 (**E**) and isotype matched IgG staining (**F**). Samples were acquired at a flow rate of 6.01 μL/min for 2 minutes. At least 5000 annexin-V^+^ CD144-PE^+^ microparticles were acquired.

Sequential gating was used to quantify the percentage of Ec-MPs among annexin-V^+^ microparticles in the urine (Fig 5). Small Angle Light Scatter (SALS), Large Angle Lights Scatter (LALS) were used to gate the microvesicle population. Annexin-V^+^ events were selected on a LALS/Green fluorescence channel plot, and the annexin-V^+^ region was defined based on unstained sample. CD144 (VE-cadherin)^+^ events in the annexin-V^+^ gate were defined on a LALS/Orange fluorescence channel and the percentage of total annexin-V^+^ microparticles was reported. Binding of isotype matched IgG was low (2.2 ±1.1%). Polymerization of uromodulin or Tamm-Horsfall protein, abundantly present in urine samples, have been reported to potentially entrap microparticles [28]. Treatment of samples with reducing agents dithiothreitol or zwitterionic detergent to release trapped microparticels showed no significant difference in the percentage Ec-MPs (% Ec-MPs: untreated: 10.0 ± 5.7; dithiothreitol treated 16.1 ± 5.7, p = 0.179, n = 5). Hematopoietic cell-derived MPs were gated in a similar way. Both annexin-V^+^ (S2 Table) and annexin-V^-^ (S3 Table) hematopoietic MPs were low in the urine samples.

### Increased endothelial microparticles in PAH and correlates with right ventricular function

**Fig 6:**
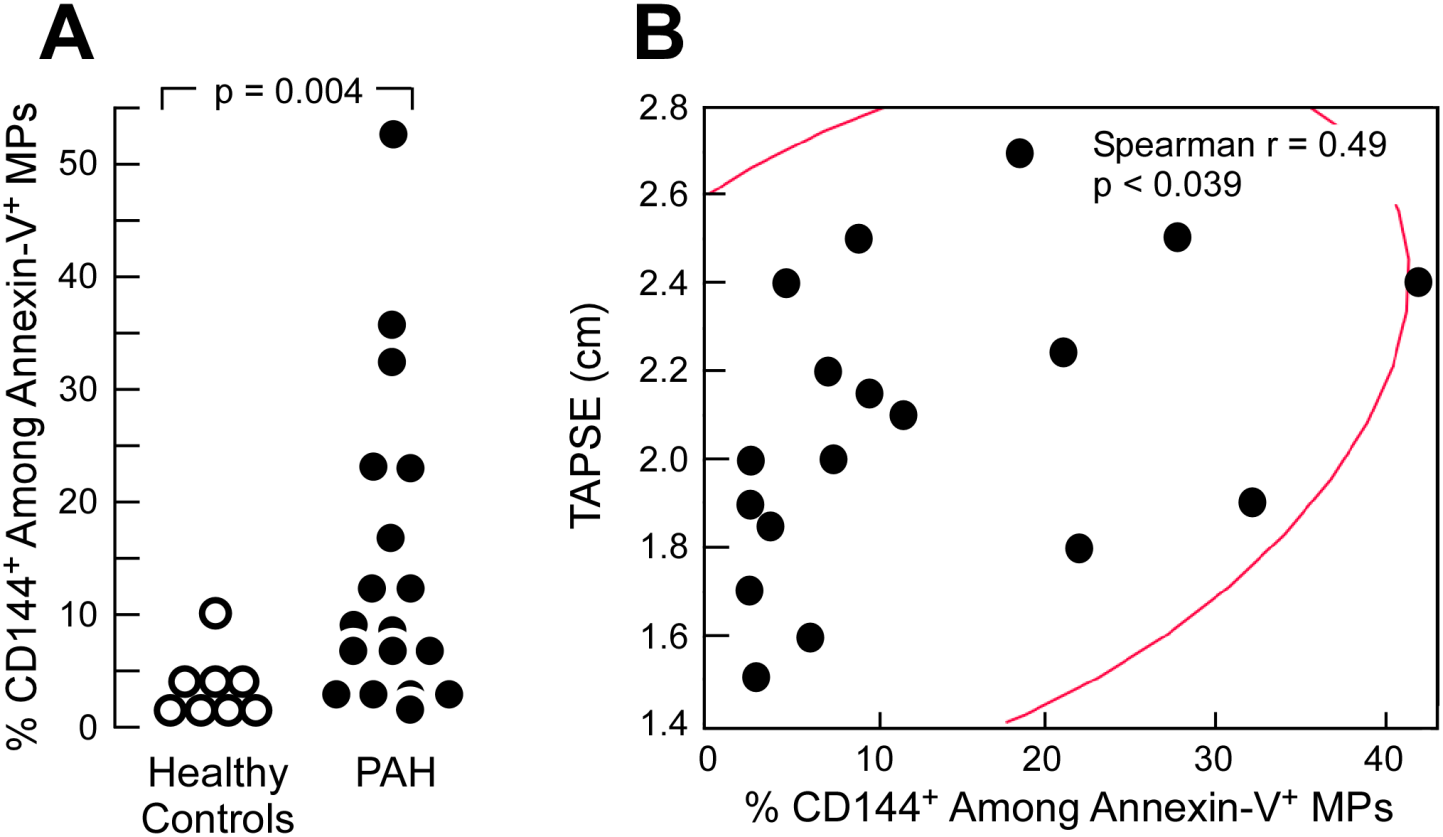
Increased endothelial microparticles in pulmonary arterial hypertension (A) and correlation with TAPSE (B). **A)** Percentage of endothelial microparticles (Ec-MPs) as defined by CD144^+^ microparticles (MPs) among annexin-V^+^ MPs was increased in PAH. p-value represents Student’s t test comparison of study groups. **B**) Percentage of Ec-MPs correlates with TAPSE. ρ represents Spearman's rank correlation coefficient with corresponding p value.

There were no significant differences in the number of MPs recovered from healthy control or PAH patients urine samples [# Annexin-V^+^ MPs/μL urine: CTRL 230 ± 131; PAH 57 ± 40, p<0.23. # Annexin-V^+^ CD144^+^ Ec-MPs//μL urine: CTRL 3.6 ± 1.6; PAH 2.6 ± 1.6, p<0.65]. The size of annexin-V^+^ CD144^+^ Ec-MPs were determined relative to the size of Apogee Mix Beads, which have a refractive index of 1.43, similar to the refractive index of biological samples (1.40) [29] [Size annexin-V^+^ CD144^+^ MPs: CTRL: 157nm, 147–176nm; PAH 142nm, 147–172nm (median, range values)]. As shown in Fig 6A, the percentage of Ec-MPs in urine were significantly elevated in PAH as compared to healthy controls (% Ec-MPs: CTRL: 2.9 ± 1.0; PAH: 14.1 ± 3.3, p<0.004). Amongst PAH patients, there was a positive correlation between % Ec-MPs and TAPSE with higher levels of % Ec-MPs correlated with higher TAPSE measurement that is associated with better right heart function (Spearman’s test ρ = 0.49, p = 0.04) (Fig 6B). The expression of CD144 on annexin-V^-^ was low, demonstrating that most Ec-MPs in the urine display the annexin-V binding phospholipid phosphatidylserine [% annexin-V^-^ CD144^+^: CTRL: 4.6 ±1.3; PAH: 2.63 ± 0.64, p<0.19]. The correlation with TAPSE and % annexin-V^-^ CD144+ was also not significant (Spearman’s test ρ = 0.38, p = 0.12).

## Discussion

There is an increasing need for biomarkers to decipher disease heterogeneity and predict treatment response in PAH. Here we report the development of new cytometric methods for assessing βAR density in peripheral blood cells with an alprenolol probe and enumeration of Ec-MPs in urine samples. Specificity of the alprenolol-probe for βAR was confirmed by demonstrating dose-dependent saturable binding that was reversed by a βAR ligand but not by a scrambled competitor losartan consistent with, classical radio-ligand studies [12]. Combination of the probe with immunophenotyping of WBC allowed the quantification of βAR density on various circulating subsets. Differences in βAR levels in peripheral blood cell subsets in general showed lower receptor levels in PAH patients compared to healthy controls. There was a trend towards βAR levels in the PAH circulating endothelial cells (CEC) correlating with TAPSE, which is a measurement of the longitudinal motion of the tricuspid annulus valve during systole. Interestingly, this has been shown to represent the function of the right ventricle and correlates strongly with prognosis in PAH [30].

The current study is a significant technical advancement over previous studies which have used leukocytes containing all the cell sub-sets to assess βAR densities. Furthermore, all the previous studies have used classical radio-ligand binding to assess βAR density in the whole leukocytes population, a method that would be very difficult to translate to large scale screening. In contrast, our method uses state of the art flow cytometry methods to differentiate the subsets of leukocyte cell populations followed by assessing βAR density in these different subsets. Since we can assess changes in βAR density within the subsets of leukocyte population, it increases our sensitivity as it is possible that the changes may occur only in one subset and not all cell types. Such an idea is supported by our observation of varying levels of βAR density within the leukocyte cell subsets. PMN had the highest level of alprenolol binding, in agreement with previous work [12], while CEC had the lowest. Interestingly, HPC had significantly higher levels than the remainder of the MNC population. A subset of HPC with proangiogenic properties have been shown to be elevated in PAH and contribute to the pathophysiology of this disease [31–33]. Previous studies have suggested that βAR receptors in lymphocytes and stem cells are involved in differentiation [34,35] or mobilization [36], and it is plausible that elevated levels in this progenitor population may play a similar role. For the most part, differences in alprenolol binding between the study groups held steady across the majority of peripheral blood cell subsets.

The finding in our study of differences in alprenolol binding between PAH patients and healthy controls aligns with previous reports of βAR dysfunction in PAH and heart failure. The study by Bristow et. al. showed lower β_1_AR density on membranes from failing right heart tissue of PAH patients as compared to matched non-failing left heart tissue from the same patient using radioligand binding studies [37]. The majority of work involving dysfunction of βAR in cardiac failure has been studied in left heart failure where sympathetic over-activity results in downregulation and desensitization of the receptor [38,39]. This process leads to reduced response to catecholamines and progressive cardiac failure [4,6]. The first studies demonstrating *β*AR dysfunction in the left heart of heart failure patients used classical radioligand binding techniques with ^3^H-dihydroalprenolol (DHA) to demonstrate decreased *β*AR density on membrane fractions of cardiac tissue [7]. It is likely that a similar mechanism underlie decreased βAR density in the failing right hearts in PAH patients. Previous studies have also shown decreased βAR density and function in lymphocytes of left heart failure patients correlate with cardiac dysfunction [9–11]. Here we show a decreased receptor density in PMNs and the total WBC population. The inability to achieve statistical significance for the other subsets is most likely a result of small sample sizes and the relatively mild disease severity of PAH patients in this study cohort; nearly 75% of patients were NYHA functional class II and mean 6 minute walk distance (MWD) was greater than 500 m. Decreased βAR density in the mature hematopoietic cells (HPC) suggests that there is likely some component of systemic effect at work in PAH affecting these circulating cells. Another explanation could involve *β*AR dysfunction in the hematopoietic stem/progenitor cell compartment that is retained during maturation. Alternatively, these cells may be responding to the local effects on passage through the pulmonary circulation.

Endothelial cell-derived microparticles are potent biomarkers cell activation, cell injury, and apoptosis [15–18]. The method for identifying microparticles in urine using flow cytometry showed that endothelial cell-derived microparticles can be detected in urine samples. This suggests that endothelial cell-derived microparticles may be released into the circulation with significant vascular dysfunction in the context of PAH and are filtered by the glomerulus, ending up in urine. Urinary extracellular vesicles derived from renal epithelial cells have been described previously in the context of renal biology [40]. However, our data show that systemic microparticles, such as endothelial cell-derived microparticles, are also present in the urine. Different studies have shown that the half-life of circulating extracellular vesicles is short within the range of 30 min to hours due to retention and uptake in different organs [40]. Our findings suggest that endothelial cell-derived microparticles are also cleared by the kidney providing a real-time window on endothelial cell function. The observations are fundamental to the idea that PAH pathology is associated with significant endothelial dysfunction and is consistent with the observation of endothelial cell-derived microparticles in the urinary samples.

In our study endothelial cell-derived microparticles correlated with levels of right ventricular function in PAH. A previous work using conventional flow cytometry showed that increased numbers of circulating endothelial cell-derived microparticles correlated to increased disease severity in PAH [17]. This may be potentially be due to the fact that different subsets of microparticles were assessed s. Conventional flow cytometers have a limit of detection (LOD) for lipid-based particles in the range of 400–800 nm [41] and there is growing evidence that >75% of extracellular vesicles are < 500 nm in [41]. The Apogee A50 Micro Flow Cytometer used in our study has a LOD of 100 nm and the Ec-MPs in urine samples were in the range of 142–176 nm and therefore, below the LOD of conventional flow cytometers. Interestingly, our data together with the work by Amabile *et al* suggest that Ec-MPs of different size may have different roles in PAH. Moreover, the art of flow cytometric extracellular vesicle detection and our knowledge of technical pitfalls have evolved substantially over the past years [23,24]. Elimination of antibody aggregates, verification of true microparticles and determination of optimal microparticle concentration to prevent swarming are critical aspects in studies of extracellular vesicles.

Interestingly, it is possible endothelial cell-derived microparticles may also vary among different patient subsets, such as patients with compensated vs decompensated disease. It is currently not known whether elevated levels of endothelial cell-derived microparticles are a cause or consequence of PAH. It may be that patients with compensated disease, endothelial cell-derived microparticles are protective and those with higher levels are better able to maintain homeostasis and right ventricular function, whereas in decompensated disease, higher levels represents a failed attempt to maintain homeostasis. Furthermore, endothelial cell-derived microparticles have been shown to both promote and inhibit angiogenesis. In fact, most studies showed that endothelial cell-derived microparticles block angiogenesis [18,19,42], which would be consistent with the finding of higher levels correlating with better right ventricle function in the TAPSE studies. Studies linking endothelial cell-derived microparticles to proangiogenic actions are most often in response to ischemic conditions [15]; apoptotic endothelial cells release microparticles that protect vasculature, stimulate the proliferation of endothelial progenitor cells and prevent endothelial-cell apoptosis showing their potential importance as communicators for survival. Endothelial cell-derived microparticles concentration appears to be an important factor determining angiogenic activity. One *in vitro* study demonstrated that microparticles isolated from human umbilical vein endothelial cells promoted angiogenesis at lower concentrations, whereas high concentrations of endothelial cell-derived microparticles suppressed angiogenesis [42]. Many other studies have shown that physiological concentrations of endothelial cell-derived microparticles do not affect angiogenesis [43–45]. Pulmonary arterial hypertension is characterized by severe angiogenic remodeling of the pulmonary arterial wall. Although endothelial cell-derived microparticles were increased in the patients, worsening of right ventricular function (decrease TAPSE) correlated with lower endothelial cell-derived microparticles, suggesting that elevated endothelial cell-derived microparticles may be protective against right ventricular dysfunction in PAH by inhibiting the pathological angiogenic remodeling.

In conclusion, the findings here show that flow cytometric quantification of peripheral blood cell βAR density and urinary endothelial cell-derived microparticles may be utilized as routine techniques to analyze clinical samples. These novel methods may be helpful as they have the power to be scaled up for easy throughputs for assessing large patient cohort to validate βAR density in circulating endothelial cells and endothelial cell-derived microparticles in urine as potential biomarkers of right ventricular function in PAH. Variability in βAR density and endothelial cell-derived microparticles may be helpful to dissect the poorly understood disease heterogeneity in PAH and predict therapy response in future studies.

## Supporting Information

S1 Fig Chemical structure and information for alprenolol-biotin probe. (DOCX)

S2 Fig Processing and staining of urine samples for endothelial cell microparticle analysis. (DOCX)

S3 Fig Sensitivity, calibration, and standardization of Apogee A50 Micro Flow Cytometer. Samples were run on an Apogee A50 Micro flow cytometer (Apogee Flow Systems, Hertfordshire, UK) equipped with a 488 nm laser. Annexin-V conjugated to AlexaFluor 488 was detected in the green channel (channel 1). VE cadherin conjugated to PE was measured in the orange channel (channel 2). The PMT values (in volts) were as follows: SALS-314, LALS-273, FL1-450, FL2-450. A threshold of 5 was applied to the LALS parameter. ApogeeMix beads (Apogee Flow Systems) ranging from sizes of 110 nm to 1300 nm with two fluorescent sizes (110 and 550 nm) were used to calibrate the sensitivity and resolution of the LALS channel. Fluorescence channels were calibrated using Spherotech rainbow calibration particles with a size of 2.08μm. A quantity of 120 uL was aspirated from each sample and ran at a flow rate of 6 μL/min. The unstained and compensation samples were run for 1 minute each and the test sample was run for 2 minutes. On average 10,000 annexin-V positive events were collected. Manual compensation was performed using Apogee Histogram software to subtract overlapping fluorescence between the green and orange channels. All microparticle data were analyzed using Apogee Histogram software.
(DOCX)

S4 Fig Annexin-V dilution curve. Samples with concentrated levels of microvesicles will result in swarming or coincidence. This is a situation during which more than a single microvescicle intercept with the laser beam at the same time. To control for this phenomenon it is critical to know that measurements are performed within a linear range. A serial dilution performed with a mixture of urine samples showed a broad linear range for the number of Annexin-V+ events/μL with our method.
(DOCX)

S1 Table Study cohort for urinary Ec-MPs. (DOCX)

S2 Table Annexin-V^+^ Hematopoietic Cell-Derived MPs in Urine. (DOCX)

S3 Table Annexin-V^-^ Hematopoietic Cell-Derived MPs in Urine. (DOCX)

## Abbreviations

*β*AR: *β*-adrenergic receptors
CEC: Circulating endothelial cells
CTRL: Healthy controls
FMO: Fluorescence minus one
HPC: Hematopoietic progenitor cells
MNC: Mononuclear cells
NYHA: New York Heart Association
PAH: Pulmonary arterial hypertension
PMN: Polymorphonuclear cells
TAPSE: Tricuspid annular plane systolic excursion
WBC: White blood cells
