# Supplementary figures and images for: Flow Cytometric Quantification of Peripheral Blood Cell β-Adrenergic Receptor Density and Urinary Endothelial Cell-Derived Microparticles in Pulmonary Arterial Hypertension
Source: PLoS One. 2016 Jun 7;11(6):e0156940. doi: 10.1371/journal.pone.0156940 (PMC4896479; doi:10.1371/journal.pone.0156940)

**
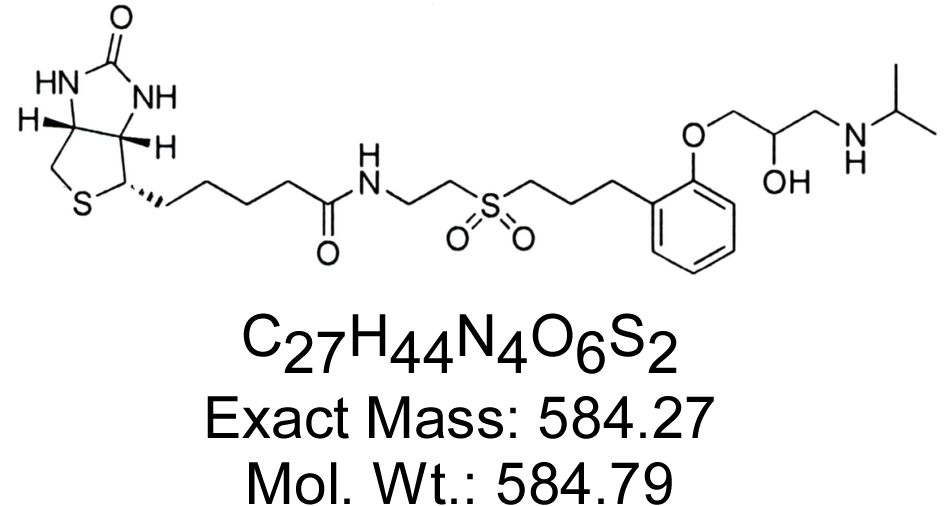
**

Supplement: S1 Fig — (DOCX) [file pone.0156940.s001.docx]

**
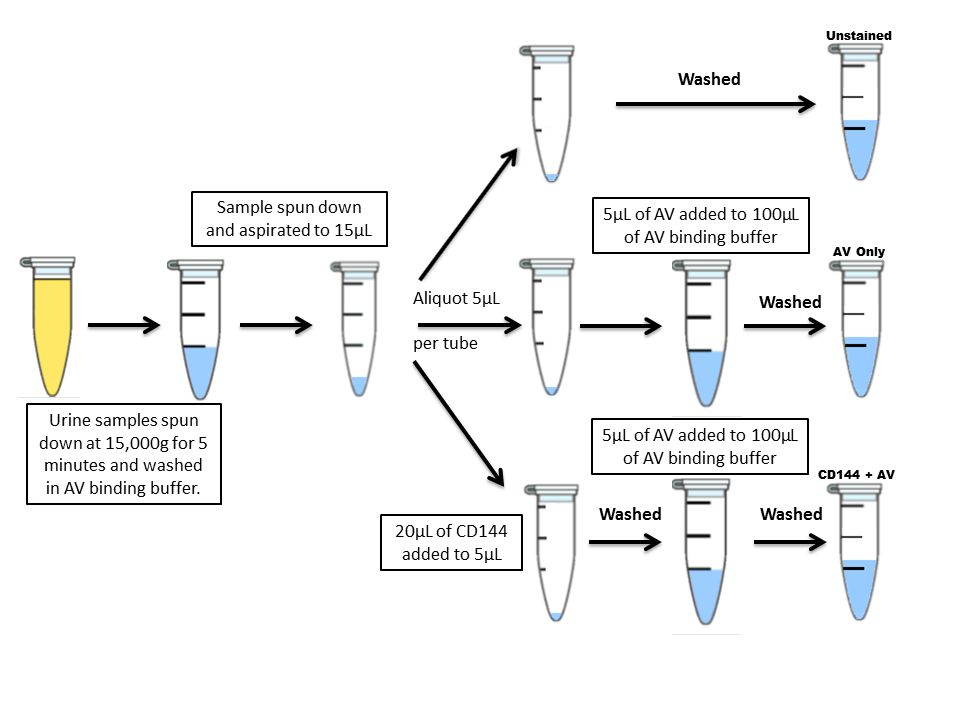
**

Supplement: S2 Fig — (DOCX) [file pone.0156940.s002.docx]

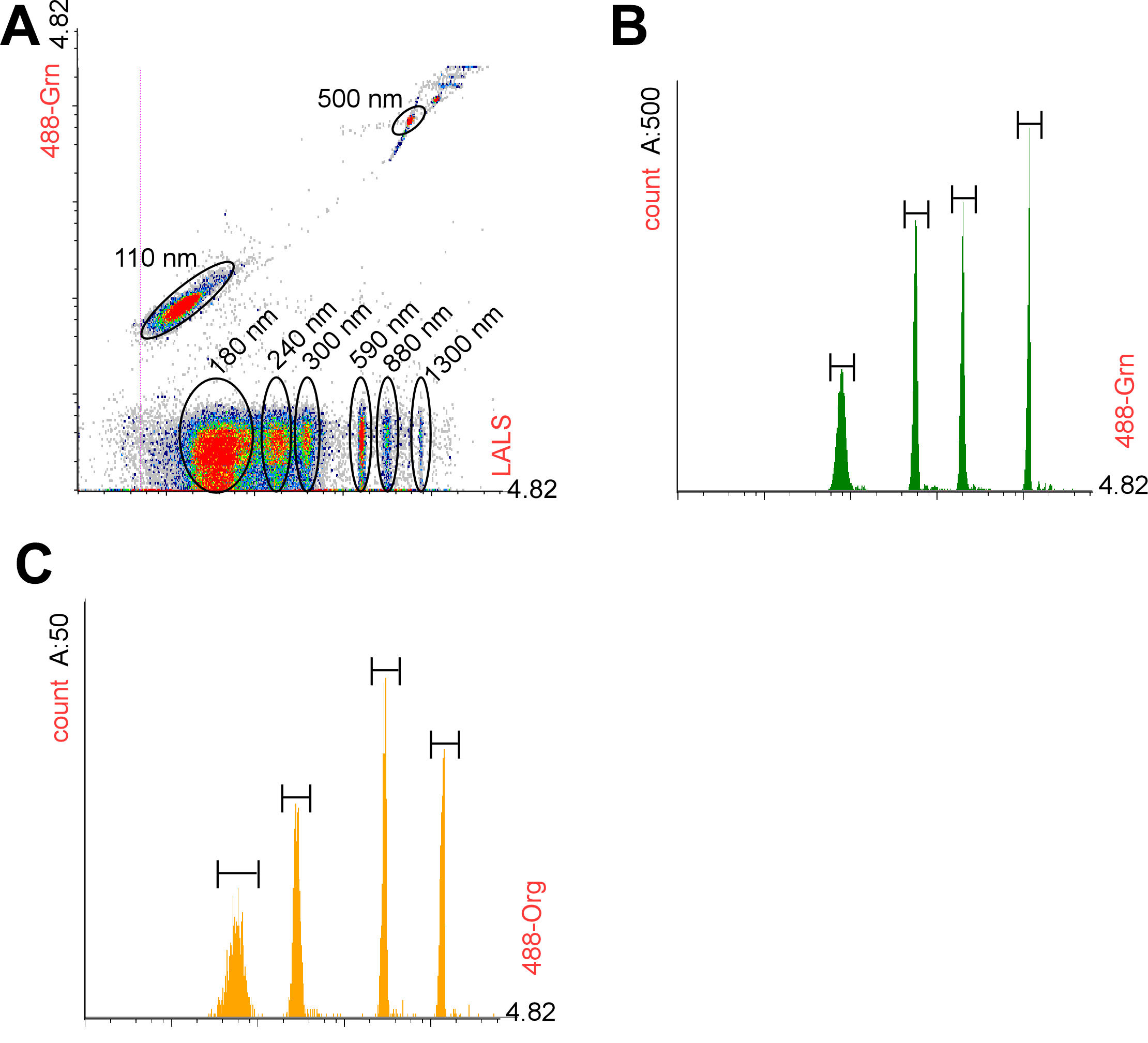

Supplement: S3 Fig — Samples were run on an Apogee A50 Micro flow cytometer (Apogee Flow Systems, Hertfordshire, UK) equipped with a 488 nm laser. Annexin-V conjugated to AlexaFluor 488 was detected in the green channel (channel 1). VE cadherin conjugated to PE was measured in the orange channel (channel 2). The PMT values (in volts) were as follows: SALS-314, LALS-273, FL1-450, FL2-450. A threshold of 5 was applied to the LALS parameter. ApogeeMix beads (Apogee Flow Systems) ranging from sizes of 110 nm to 1300 nm with two fluorescent sizes (110 and 550 nm) were used to calibrate the sensitivity and resolution of the LALS channel. Fluorescence channels were calibrated using Spherotech rainbow calibration particles with a size of 2.08μm. A quantity of 120 uL was aspirated from each sample and ran at a flow rate of 6 μL/min. The unstained and compensation samples were run for 1 minute each and the test sample was run for 2 minutes. On average 10,000 annexin-V positive events were collected. Manual compensation was performed using Apogee Histogram software to subtract overlapping fluorescence between the green and orange channels. All microparticle data were analyzed using Apogee Histogram software. (DOCX) [file pone.0156940.s003.docx]

**
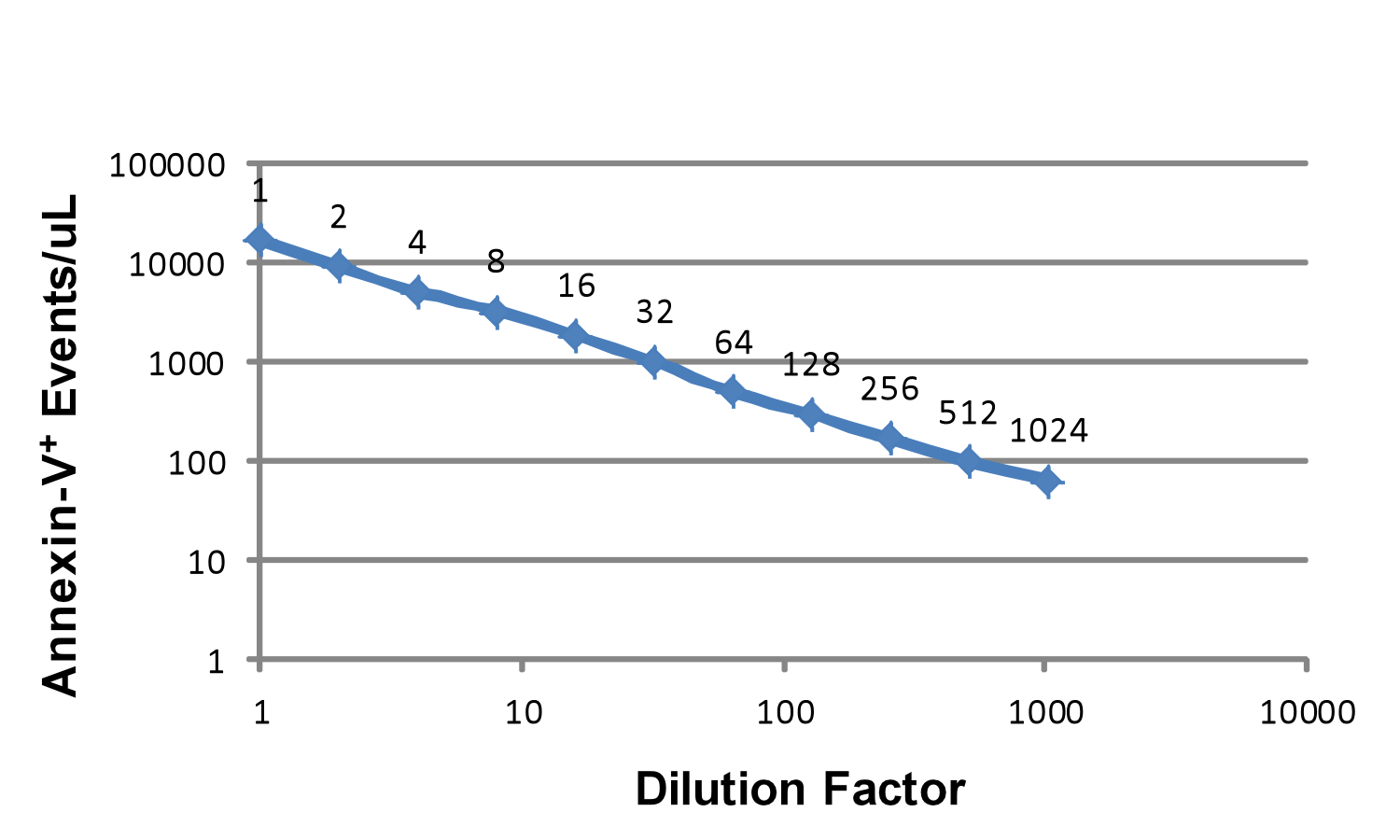
**

Supplement: S4 Fig — Samples with concentrated levels of microvesicles will result in swarming or coincidence. This is a situation during which more than a single microvescicle intercept with the laser beam at the same time. To control for this phenomenon it is critical to know that measurements are performed within a linear range. A serial dilution performed with a mixture of urine samples showed a broad linear range for the number of Annexin-V+ events/μL with our method. (DOCX) [file pone.0156940.s004.docx]
