# Supplementary material for: Flow Cytometric Quantification of Peripheral Blood Cell β-Adrenergic Receptor Density and Urinary Endothelial Cell-Derived Microparticles in Pulmonary Arterial Hypertension
Source: PLoS One. 2016 Jun 7;11(6):e0156940. doi: 10.1371/journal.pone.0156940 (PMC4896479; doi:10.1371/journal.pone.0156940)
Supplement: S1 Table — (DOCX) [file pone.0156940.s005.docx]

|  | CTRL | PAH |
| --- | --- | --- |
| Number | 8 | 18 |
| Age (years), mean ± SD | 37.0 ± 4.7 | 46.5 ± 2.7 |
| Gender (% female) | 25% | 56% |
| Race (C/AA/), N (%) | 2(25%)/6(75%) | 15(83%)/3(17%) |
| 6MWD (ft), mean ± SD | - | 1543 ± 97 |
| RVSP (mmHg), mean ± SD | - | 66.7 ± 6.95 |
