# Supplementary material for: Flow Cytometric Quantification of Peripheral Blood Cell β-Adrenergic Receptor Density and Urinary Endothelial Cell-Derived Microparticles in Pulmonary Arterial Hypertension
Source: PLoS One. 2016 Jun 7;11(6):e0156940. doi: 10.1371/journal.pone.0156940 (PMC4896479; doi:10.1371/journal.pone.0156940)
Supplement: S2 Table — (DOCX) [file pone.0156940.s006.docx]

| **% AV^+^ MPs Subset** | **CTRL** | | | **PAH** | | |
| --- | --- | --- | --- | --- | --- | --- |
|  | **Mean** | **±** | **SE** | **Mean** | **±** | **SE** |
| **CD3** | 4.58 | ± | 2.315 | 0.19 | ± | 0.058 |
| **CD19** | 0.112 | ± | 0.054 | 0.072 | ± | 0.02 |
| **CD34** | 0.082 | ± | 0.052 | 0.118 | ± | 0.032 |
| **CD45** | 0.864 | ± | 0.584 | 0.134 | ± | 0.055 |
| **CD133** | 0.17 | ± | 0.108 | 0.076 | ± | 0.014 |
