# Supplementary material for: Flow Cytometric Quantification of Peripheral Blood Cell β-Adrenergic Receptor Density and Urinary Endothelial Cell-Derived Microparticles in Pulmonary Arterial Hypertension
Source: PLoS One. 2016 Jun 7;11(6):e0156940. doi: 10.1371/journal.pone.0156940 (PMC4896479; doi:10.1371/journal.pone.0156940)
Supplement: S3 Table — (DOCX) [file pone.0156940.s007.docx]

| **% AV^-^ MPs Subset** | **CTRL** | | | **PAH** | | |
| --- | --- | --- | --- | --- | --- | --- |
|  | **Mean** | **±** | **SE** | **Mean** | **±** | **SE** |
| **CD3** | 0.462 | ± | 0.081 | 0.366 | ± | 0.081 |
| **CD19** | 0.134 | ± | 0.023 | 0.158 | ± | 0.027 |
| **CD34** | 0.304 | ± | 0.094 | 0.242 | ± | 0.032 |
| **CD45** | 0.27 | ± | 0.061 | 0.186 | ± | 0.038 |
| **CD133** | 0.174 | ± | 0.079 | 0.186 | ± | 0.035 |
